# CCN1 in hepatobiliary injury repair

**DOI:** 10.18632/oncotarget.6079

**Published:** 2015-10-11

**Authors:** Ki-Hyun Kim, Lester F. Lau

**Affiliations:** Department of Biochemistry and Molecular Genetics, University of Illinois at Chicago, Chicago, IL, USA

**Keywords:** wound healing, cholestasis, hepatocellular carcinoma, fibrosis, senescence

CCN1 (CYR61) is a secreted matricellular protein capable of regulating various cellular activities through interaction with distinct integrin receptors in a cell type-and context-dependent manner [1]. Although CCN1 is essential for cardiovascular development during embryogenesis, in adulthood its functions are associated with inflammation and wound healing [2]. Several recent studies have identified surprisingly diverse yet critical roles of CCN1 in multiple aspects of hepatobiliary injury repair, including bile duct regeneration, resolution of liver fibrosis, and suppression of carcinogen-induced hepatocellular carcinoma (HCC). These functions are mediated through distinct mechanisms targeting disparate cell types, underscoring the versatility of matricellular signaling through various integrin receptors.

Liver fibrosis occurs as a wound-healing response to chronic hepatic injuries, irrespective of the underlying etiology, and may progress to life-threatening cirrhosis. Although deposition of extracellular matrix (ECM) can promote injury repair by enhancing tissue integrity and cell proliferation, chronic injuries often result in excessive ECM accumulation, leading to fibrosis and loss of organ function. The liver is a regenerative organ and summons robust repair mechanisms upon injury. The repair response to biliary injuries includes cholangiocyte proliferation, expansion of bile ducts, and differentiation of hepatic progenitor cells (HPCs) into cholangiocytes. A recently publication has shown that CCN1 is greatly elevated in hepatocytes and cholangiocytes in response to hepatic injuries, and is indispensable for biliary regeneration [3]. CCN1 induces bile duct expansion through engagement of integrins α_v_β_5_ and α_v_β_3_ in cholangiocytes, thereby activating NFκB to induce *Jag1* expression, leading to Jag1/Notch signaling and cholangiocyte proliferation [3]. In addition, CCN1 also induces *Jag1* expression in hepatic stellate cells, which drive the differentiation of HPCs into cholangiocytes. Consistent with an essential role for CCN1-α_v_β_5_/α_v_β_3_ interaction in biliary repair, knockin mice (*Ccn1^D125A/D125A^*) expressing a CCN1 mutant with a single amino acid substitution that disrupts its α_v_β_5_/α_v_β_3_-binding site are severely impaired in bile duct regeneration, resulting in massive hepatic necrosis and rapid mortality upon cholestasis induced by bile duct ligation (BDL). By contrast, wild type mice survive well within the same experimental period owing to expanded bile ducts that shield bile acids from the parenchyma. Wild type mice suffer defects similar to the knockin mice in bile duct regeneration after BDL when subjected to blockade of integrins α_v_β_5_/α_v_β_3_, NFκB activation, or Notch signaling, whereas activation of Jag/Notch signaling in livers of *Ccn1^D125A/D125A^* mice ameliorates the mutant phenotypes [3]. These findings identify CCN1-α_v_β_5_/α_v_β_3_-NFκB-Jag1 as a critical axis for biliary injury repair.

Remarkably, CCN1 also functions to dampen and limit liver fibrosis. In fibrogenesis due to hepatotoxic and cholestatic injuries, hepatic stellate cells and portal fibroblasts, respectively, are the major precursor cells that transdifferentiate into myofibroblasts responsible for the production of ECM. CCN1 restricts and resolves fibrosis by triggering senescence in these myofibroblasts, whereupon the senescent cells express an anti-fibrotic program that includes secretion of matrix-degrading enzymes, thus converting these ECM-producing cells into ECM-degrading cell [2, 4]. Mechanistically, CCN1 acts by engaging integrin α_6_β_1_ to induce sustained accumulation of reactive oxygen species (ROS) through the RAC1-NADPH oxidase 1 complex, triggering senescence. Mice with hepatocyte-specific deletion of *Ccn1* (*Ccn1^ΔHep^*) or knockin mice expressing an α_6_β_1_-binding defective CCN1 mutant (*Ccn1^dm/dm^*) sustain exacerbated fibrosis with concomitant deficit in myofibroblast senescence, whereas overexpression of *Ccn1* in hepatocytes reduces liver fibrosis with enhanced senescence [4]. Moreover, tail vein delivery of purified CCN1 protein in mice accelerates resolution of established fibrosis and adenoviral expression of *Ccn1* attenuates BDL-induced liver fibrosis [4, 5], further establishing the anti-fibrotic effects of CCN1.

Yet another activity of CCN1 inhibits the formation of HCC during hepatic injury repair. Hepatocarcinogens such as diethylnitrosoamine (DEN) induce HCC by causing DNA damage and apoptosis in hepatocytes, thus triggering hepatocyte proliferation to compensate for the cell loss. However, this compensatory proliferation may stimulate the expansion of damaged and mutated hepatocytes that are at risk of oncogenic transformation, thereby promoting carcinogenesis. CCN1 suppresses DEN-induced HCC by inhibiting epidermal growth factor receptor (EGFR)-dependent hepatocyte compensatory proliferation through integrin α_6_β_1_, leading to ROS-dependent activation of p53 and cell cycle block [6]. Consequently, *Ccn1^ΔHep^* and *Ccn1^dm/dm^* mice exhibit elevated tumor multiplicity in DEN-induced HCC. Furthermore, a single dose of the EGFR inhibitor erlotinib delivered prior to DEN exposure blocked compensatory proliferation and obliterated HCC formation, indicating that the CCN1-inhibitable EGFR-dependent hepatocyte compensatory proliferation play a critical role in the development of HCC [6].

These findings reveal key functions of CCN1 in diverse aspects of liver injury repair ranging from biliary regeneration, resolution of fibrosis, and suppression of hepatocarcinogenesis through distinct mechanisms. Moreover, recent discovery shows that CCN1 enhances the resolution of inflammation by promoting phagocytic clearance of apoptotic neutrophils [7], and this activity may also play a role in hepatic wound repair. Further studies are needed to elucidate the possible functions of CCN1 in other modes of hepatic injuries, including nonalcoholic steatohepatitis, viral infections, autoimmune disorders, and in repair mechanisms that may invoke the participation of HPCs. Insights into the diverse functions of CCN1 in hepatobiliary injury repair may prompt explorations on their potential therapeutic value in treating chronic liver diseases.
